# Importance of Continuous Pulse Oximetry of the Ipsilateral Thumb/Index Finger during Transradial Angiography

**DOI:** 10.1155/2011/653625

**Published:** 2011-12-14

**Authors:** Ross C. Puffer, David F. Kallmes

**Affiliations:** ^1^Mayo Medical School, Mayo Clinic, Rochester, MN 55905, USA; ^2^Department of Radiology, Mayo Clinic, Rochester, MN 55905, USA

## Abstract

We present a case of a 63-year-old male undergoing attempted basilar artery embolization using a right transradial artery approach in which continuous pulse oximetry of the ipsilateral thumb uncovered unanticipated hand ischemia during the procedure. A preprocedural evaluation using pulse oximetry of the right thumb demonstrated normal waveform and maintenance of normal oxygen saturation during manual compression of the right radial artery. This normal waveform and oxygen saturation was maintained after insertion of a 6Fr sheath into the radial artery. After insertion of a 6Fr guiding catheter into the right vertebral artery, near-complete dampening of the pulse oximetry waveform and precipitous decline in oxygen saturation were noted. Examination of the right hand demonstrated poor tissue perfusion. Immediate removal of the guiding catheter led to return of a normal waveform, oxygen saturation, and tissue perfusion. This case demonstrates the importance of continuous, intraprocedural monitoring of oxygenation of the ipsilateral hand during transradial angiography in order to avoid unanticipated perfusion abnormalities.

## 1. Introduction

One of the most serious complications of the transradial approach is radial artery occlusion secondary to lack of sufficient collateral flow through the ulnar artery and palmar arch [1–5]. This is also the most common complication, having an incidence of 2–18% depending on techniques used [2–5]. Numerous maneuvers have been proposed to assess risk for inadequate tissue perfusion with radial artery occlusion. The most well known of these maneuvers is the Allen test, but the subjective nature of this test limits its efficacy. Pulse oximetry of the ipsilateral thumb or index finger during manual compression of the radial artery has been shown to correlate with Doppler imaging in its ability to characterize ulnar collateral flow, making it a more accurate and superior evaluation tool as compared to the Allen test [6–8]. While pulse oximetry of the ipsilateral thumb or index finger is commonly used as a *preprocedure* evaluation technique, we have found *continuous, intraprocedural* pulse oximetry monitoring of the ipsilateral thumb or index finger useful to avoid ischemic complications. In order to illustrate this point, we offer a case report in which intraprocedural pulse oximetry identified unanticipated ischemia of the ipsilateral hand during transradial angiography.

## 2. Case

A 63-year-old man presented to our institution for endovascular treatment of a large, right vertebrobasilar aneurysm. Prior to radial artery catheterization, a pulse oximeter was placed on the right thumb and a stable waveform and oxygen saturation were obtained. During manual compression of both the right radial and ulnar artery, complete damping of waveform was noted. Upon release of the ulnar arterial compression, a robust waveform, identical to the baseline waveform, returned immediately, along with normal oxygen saturation. The pulse oximeter was left in place on the right thumb.

Using a transradial approach, a 6Fr sheath was placed, with maintenance of a normal waveform. A 6Fr guiding catheter (Envoy) was placed into the right vertebral artery. Several minutes later, the nurse anesthetist alerted the physician that the waveform had flattened substantially and that the oxygen saturation dropped precipitously. Examination of the right hand demonstrated poor capillary refill. The guiding catheter was removed and a normal waveform returned to the pulse oximeter, associated with normal oxygen saturation. The case was completed from a transfemoral approach. Following the basilar artery embolization from the femoral approach, the radial and femoral sheaths were removed. The right hand remained normal, without evidence for ischemic complications.

## 3. Discussion

We routinely apply continuous, intraprocedural pulse oximetry of the ipsilateral thumb or index finger, as opposed to preprocedure monitoring alone, to determine adequate perfusion of the hand during interventional procedures in which transradial access is attempted. The case that we present here demonstrates that even when preprocedure pulse oximetry confirms that flow through the ulnar artery is sufficient to perfuse the hand when the radial artery is completely occluded, various techniques and manipulations used during the procedure may induce hand ischemia. We presume that in our case, the guiding catheter impeded flow in an important collateral vessel and that prompt removal of the catheter thus restored adequate collateral flow. Without prompt identification and treatment of hand ischemia, thrombotic or ischemic complications might have occurred. Thus, we conclude that it is important to monitor the perfusion of the hand not only in the preprocedure setting but also throughout the duration of the intervention to ensure that the hand is not at risk for ischemic damage.

The transradial approach is being used more often in the setting of vascular intervention, and common complications attributable to this technique are becoming better understood. We are not aware of any previous studies demonstrating the importance of intraoperative pulse oximetry of the ipsilateral thumb or index finger to monitor perfusion of the hand, even though preprocedural pulse oximetry is routine in many institutions [9]. Further, numerous, previous studies have shown the serious effects of hand ischemia in the setting of radial artery occlusion without adequate collateral flow through the ulnar artery and palmar arch, highlighting the importance of tissue perfusion during and after transradial angiography [1, 3, 5, 10–16].

We realize that this finding was observed in a single patient and that we have not shown the incidence of this complication in a large series. Even so, we believe that intraoperative monitoring is an excellent method of early detection of hand ischemia and that through the use of this monitoring technique, further studies may uncover the true incidence of this complication.

## 4. Conclusions

Using pulse oximetry to monitor blood flow to the hand during a transradial angiographic procedure is an efficient method to prevent silent ischemia from occurring during the operation.
